# Impact of COVID-19 outbreak on hospital admissions and outcome of acute coronary syndromes in a single high-volume centre in southeastern Europe

**DOI:** 10.1007/s12471-021-01554-x

**Published:** 2021-03-11

**Authors:** M. Petrović, A. Milovančev, M. Kovačević, T. Miljković, A. Ilić, A. Stojšić-Milosavljević, M. Golubović

**Affiliations:** 1 Faculty of Medicine, University of Novi Sad, Novi Sad, Serbia; 2 Institute of Cardiovascular Diseases of Vojvodina, Sremska Kamenica, Serbia

**Keywords:** COVID-19, Acute coronary syndrome, Mortality

## Abstract

**Background:**

As coronavirus disease 2019 (COVID-19) has reached pandemic status, authors from the most severely affected countries have reported reduced rates of hospital admissions for patients with acute coronary syndrome (ACS).

**Aim:**

The aim of the present study was to investigate the influence of the COVID-19 outbreak on hospital admissions and outcomes in ACS patients in a single high-volume centre in southeastern Europe.

**Methods:**

This retrospective observational study aimed to investigate the number of hospital admissions for ACS, clinical findings at admission, length of hospitalisation, major complications and in-hospital mortality during the COVID-19 outbreak and to compare the data with the same parameters during an equivalent time frame in 2019. For the ST-elevated myocardial infarction (STEMI) subgroup of patients, changes in ischaemic times were analysed as well.

**Results:**

There was a significant reduction of 44.3% in the number of patients admitted for ACS during the COVID-19 outbreak when compared with the same period in 2019 (151 vs 271; 95% confidence interval 38.4–50.2, *p* < 0.01) with a higher mortality rate (13.2% vs 7.2%, *p* = 0.03). In 2020, patients with non-ST-elevated myocardial infarction presented more often with acute heart failure (3.3% vs 0.7%, *p* = 0.04). During the COVID-19 outbreak, we observed increases in the total ischaemic time (303 ± 163.4 vs 200.8 ± 156.8 min, *p* < 0.05) and door-to-balloon time (69.2 ± 58.4 vs 50.5 ± 31.3 min, *p* < 0.01) in STEMI patients.

**Conclusions:**

These findings should increase the awareness of morbidity and mortality related to missed or delayed treatment of ACS among the public and the healthcare services.

## What’s new?

- Restrictive measures during the coronavirus disease 2019 (COVID-19) outbreak in Serbia considerably affected the hospital admission and treatment of patients with acute coronary syndromes (ACS).
- During the COVID-19 outbreak in Serbia, mortality and complication rates for ACS patients were significantly higher, as were patient-related times (from symptom onset to first medical contact) and system-related times (from first medical contact until reperfusion), but hospital admissions for ACS decreased substantially.

## Introduction

The number of people infected with severe acute respiratory syndrome coronavirus 2 (SARS-CoV-2) has recently increased rapidly. Governments worldwide are proposing more restrictive measures. The Serbian government introduced restrictive measures from 16 March until 6 May 2020 to control the spread of the coronavirus disease 2019 (COVID-19) outbreak. The entire national health care system was reorganised to control the current pandemic. We have no data on how these measures have affected the diagnosis and treatment of other acute medical conditions, such as acute coronary syndrome (ACS).

To date, different authors from the most severely affected countries have reported reductions in hospital admissions for ACS during the COVID-19 pandemic [1–6]. To the best of our knowledge, data from southeastern European centres are missing. The aim of the present study was to investigate the impact of the COVID-19 outbreak on hospital admissions and mortality in ACS patients in our high-volume centre in Serbia.

## Methods

We performed a retrospective observational study aiming to evaluate hospital admissions for ACS during the COVID-19 outbreak (over the course of a 7-week period from 16 March to 6 May 2020) and compared the data with the same parameters during an equivalent time frame in 2019.

We included all consecutive patients who were admitted to the Cardiology Department of the Institute of Cardiovascular Diseases of Vojvodina (ICVDV), Serbia, in a predefined time frame. Our regional university centre is the hub of the Vojvodina ST-elevation myocardial infarction (STEMI) network, providing care to 2 million inhabitants (approximately one quarter of the entire Serbian population) and performing approximately 1300 primary percutaneous coronary interventions (pPCIs) per year, and therefore constitutes a high-volume centre.

All consecutive patients admitted for ACS, STEMI, non-ST-segment elevation myocardial infarction (NSTEMI) and unstable angina (UA) were included in the survey. Acute myocardial infarction (AMI) was defined in accordance with the Fourth Universal Definition of Acute Myocardial Infarction [7].

We collected and analysed data on patient risk factors, comorbidities, clinical findings at admission, length of hospitalisation, major complications, ejection fraction (EF) at discharge and in-hospital mortality. Major complications were defined as mechanical complications (free wall rupture, postinfarction ventricular septal defect or severe functional mitral regurgitation), life-threatening arrhythmias and cardiogenic shock. Killip classes III and IV were considered acute heart failure.

For the STEMI subgroup of patients, we calculated ischaemic times as well, particularly the time from symptom onset until hospital admission, door-to-balloon time (the time from presentation at the emergency room until the first balloon inflation during PCI) and total ischaemic time; these times were calculated for STEMI patients who underwent PCI within 12 h of symptom onset.

These parameters were extracted from the electronic records for the same time frame in 2019 and 2020 and compared. Data were finally checked by one investigator for missing or contradictory entries and for values outside of the normal range. The study was conducted according to the principles of the Declaration of Helsinki. The study was approved by the Ethics Committee of the ICVDV.

### Statistical analysis

Continuous variables are presented as the means and standard deviations, and categorical variables are presented as absolute numbers and percentages. Differences were tested by Student’s *t*-test and the chi-square test. The risk ratio (RR) was calculated with the 95% confidence interval (95% CI) and compared by the chi-square test. Values lower than 0.05 were considered significant. The statistical software Statistic (Statistic 13.5, The Ultimate Academic Bundle, StatSoft Europe GmbH, Hamburg, Germany; university licence for the University of Novi Sad) was used for all analyses.

## Results

A total of 422 patients with ACS (151 in 2020 during the COVID-19 outbreak and 271 in the same period in 2019) were included in the study. The clinical characteristics and comorbidities of patients admitted for ACS were similar in the two years (Tab. 1). There were significantly more patients with previous PCI and a sedentary lifestyle in 2020 than in 2019 (4.4% vs 11.3%, *p* = 0.007; 0.7% vs 3.9%, *p* = 0.01, respectively).

**Table 1:** Characteristics of patients with acute coronary syndrome (*ACS*)

Characteristics	2019 (*n* = 271)	2020 (*n* = 151)	*p*-value
History of hypertension	154 (57%)	89(59%)	NS
History of diabetes	54 (20%)	31(21%)	NS
History of hyperlipidaemia	50 (18%)	38(25%)	NS
History of chronic kidney disease	6 (2.2%)	5 (3.3%)	NS
Family history of CVD	57 (21%)	38 (25%)	NS
Smoking history	99 (36.5%)	67 (44.4%)	NS
Alcohol history	3 (1.1%)	3 (3.2%)	NS
Sedentary lifestyle	2 (0.7%)	6 (3.9%)	0.01
Stress	5 (1.85%)	4 (2.6%)	NS
COPD	5(1.85%)	5 (3.3%)	NS
Malignancy	6 (2.2%)	9 (5.2%)	NS
Peptic ulcer disease	3 (1.1%)	1 (0.7%)	NS
Anaemia	3 (1.1%)	1 (0.7%)	NS
Prior stroke	8 (2.9%)	6 (3.9%)	NS
Prior MI	25 (9.2%)	21(13.9%)	NS
Prior PCI	12 (4.4%)	17(11.3%)	0.007
Prior CABG	5 (1.8%)	7 (4.6%)	NS
Prior pacemaker	1 (0.4%)	0	NS
*Clinical examination characteristics on admission*			
BMI (kg/m^2^)	27.7 ± 5.5	28.7 ± 4.9	NS
Blood pressure systolic (mm Hg)	138.1 ± 28.8	136 ± 28.4	NS
Blood pressure diastolic (mm Hg)	80.6 ± 15.9	79.6 ± 16.8	NS
Heart rate/min	82 ± 19	80 ± 21	NS
Killip I and II	241 (88.9%)	132 (87.4%)	NS
Killip III and IV	30 (11.1%)	19 (12.6%)	NS

*BMI* body mass index, *CABG* coronary artery bypass grafting, *COPD* chronic obstructive pulmonary disease, *CVD* cardiovascular disease, *MI* myocardial infarction, *PCI* percutaneous coronary intervention

There was a significant reduction of 44.3% in the number of patients admitted for ACS during the COVID-19 outbreak in 2020 than in the same period in 2019 (151 vs 271; 95% CI 38.4–50.2, *p* < 0.01) (Tab. 2). There was no significant difference between 2019 and 2020 in the mean patient age (64.8 ± 11.9 vs 65.2 ± 11.3 years; *p* = 0.703) or gender distribution [males 192 (70.8%) vs 97 (64.2%); *p* = 0.1].

**Table 2:** Reduction in hospital admissions, in-hospital mortality and complications in patients with acute coronary syndrome (*ACS*) before (2019) and during (2020) the COVID-19 outbreak

	2019				2020				
%	All ACS	STEMI	NSTEMI	UA	All ACS	STEMI	NSTEMI	UA	*p*-value
Reduction in admissions *n* (%) (95% CI)	271* (44.3%) (38.4–50.2)				151*				<0.01*
		186^**^ (38.7%) (31.7–46.1)				114^**^			<0.01^**^
			56^***^ (55.4%) (31.3–58.5)				31^***^		<0.01^***^
				29^****^ (79.3%) (60.3–92.0)				6^****^	<0.01^****^
In-hospital mortality	7.7*				13.2*				0.03*
		7.5^**^				14.9^**^			0.02^**^
			9.7^***^				10.7^***^		0.7^***^
				3.5^****^				0^****^	0.002^****^
AHF	11.1*			–	12.6*				0.6*
		4.4^**^				7.2^**^			0.2^**^
			0.7^***^				3.3^***^		0.04^***^
EI	3.7				10.6				0.04
Major complications	13.3			–	15.9				0.3

*STEMI* ST-segment elevated myocardial infarction, *NSTEMI* non-ST-segment elevated myocardial infarction, *UA* unstable angina, *AHF* acute heart failure (Killip class III and IV), *EI* endotracheal intubation, *CI* confidence interval

*p*-value related to *ACS, ^**^STEMI, ^***^NSTEMI, ^****^UA

Comparing the ACS subgroups, the reduction in hospital admissions was highest for UA (79.3%, 95% CI 60.3–92.0, *p* < 0.01), followed by NSTEMI (55.4% reduction, CI 31.3–58.5, *p* < 0.01) and STEMI (38.7% reduction, 95% CI 31.7–46.1, *p* < 0.01).

In 2020, patients with ACS presented more often with acute heart failure (all ACS 12.6% vs 11%, *p* = 0.6; STEMI 7.2% vs 4.4%, *p* *=* 0.2; NSTEMI 3.3 vs 0.7%, *p* = 0.04) and major complications (all ACS patients 15.9% vs 13.3% in 2019, *p* = 0.3). The need for endotracheal intubation and invasive mechanical ventilation increased significantly in 2020 (10.6% vs 3.7%, *p* = 0.04).

The length of hospitalisation for all ACS patients was significantly longer in 2019 (6.9 ± 7.4 vs 4.1 ± 2.7 days, *p* < 0.0001) than in 2020, and overall in-hospital mortality increased from 7.7% in 2019 to 13.2% in 2020 [*p* = 0.03; RR = 1.71 (95% CI 0.957–3.05), *p* = 0.06]. All ACS patients included in the analysis were negative for SARS-CoV‑2 based on the results of the quantitative fluorescence-based reverse transcription polymerase chain reaction.

**Fig. 1:**
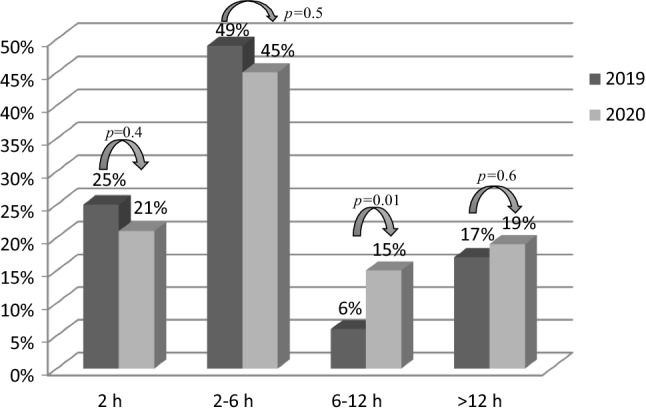
Time from onset of symptoms until hospital admission for patients with ST-elevation myocardial infarction before (2019) and during (2020) the COVID-19 outbreak

Concerning the STEMI subgroup of patients, late presentation (6–12 h from symptom onset) increased significantly during the COVID-19 outbreak (14.7% vs 6%, *p* = 0.01). Very late STEMI presentation (> 12 h from symptom onset) increased from 17% to 19.3% (*p* = 0.6; Fig. 1). The average door-to-balloon time was significantly higher in 2020 than in 2019 (69.2 ± 58.4 vs 50.5 ± 31.3 min, *p* < 0.01), as was the total ischaemic time (303 ± 163.4 vs 200.8 ± 156.8 min, *p* < 0.05). PCI rates among STEMI patients were comparable (89.8% vs 86%, *p* = 0.2) between the two years.

The STEMI mortality rate during the COVID-19 outbreak increased substantially to 14.9% compared with 7.5% in 2019 [*p* = 0.02; RR = 1.98 (95% CI 1.016–3.86), *p* = 0.04]. The length of hospital stay was significantly shorter in 2020 for STEMI patients (4.2 ± 2.8 vs 5.5 ± 3.6 days, *p* < 0.001). The EF on discharge was lower for STEMI patients in 2020 (44.9 ± 10.7% vs 49.3 ± 9.4%, *p* = 0.18).

The NSTEMI and UA patients admitted in 2020 had significantly higher PCI rates (80% vs 51.8%, *p* < 0.001; 45% vs 83.3%, *p* < 0001, respectively) than those admitted in 2019. The length of hospitalisation was significantly shorter for both NSTEMI (10.9 ± 12.7 to 4.1 ± 2.3 days, *p* = 0.004) and UA (9.3 ± 8.2 to 2.5 ± 1.2 days, *p* = 0.05) patients during the COVID-19 outbreak. The mortality rate in the NSTEMI group was 9.7% in 2019 and 10.7% in 2020 [*p* = 0.44; RR = 0.9032 (95% CI 0.24–3.36), *p* = 0.879].

### Discussion

The main finding of the present study was a significant (44.3%) reduction in the number of hospitalisations for ACS and an increased mortality rate in STEMI patients during the COVID-19 outbreak in Serbia. The reduction in admissions for UA and NSTEMI was higher than that for the STEMI subgroup. These results are in line with the findings reported in similar studies.

In December 2019, China reported the first cluster of cases of pneumonia caused by a new virus in Wuhan. In January 2020, the World Health Organisation published the first news on the disease outbreak. In late January and February, investigators from Hong Kong [8] observed large delays in the treatment of the small number of patients with STEMI. As the COVID-19 outbreak evolved into a pandemic, more authors have reported the impact of the outbreak on patients with ACS.

Metzler et al. [9] reported a 39.4% reduction in hospitalisations for ACS (greater for NSTEMI than for STEMI) in Austria. Several authors from Italy, one of the countries most severely affected by the COVID-19 pandemic, reported a reduced hospitalisation rate for ACS patients. De Filippo et al. [10] reported that hospitalisations for ACS (STEMI, NSTEMI and UA) decreased by 39.2% compared to the pre-COVID-19 period in 2020 and by 27.6% compared to 2019. De Rosa et al. [1] reported a dramatic reduction (48.4%) in the number of hospitalisations for AMI (greater for NSTEMI than for STEMI) across Italy during the COVID-19 pandemic. Secco et al. [2] reported a 48.1% reduction in hospitalisations for AMI (greater for NSTEMI than for STEMI). Toniolo et al. [4] showed a 52.1% reduction in the number of hospitalisations for ACS (greater for NSTEMI than STEMI).

Patients with prior PCI may be able to recognise the symptoms of myocardial ischaemia better and were significantly more prevalent in 2020 in our research.

During the COVID-19 outbreak in Serbia, patients tended to come to the hospital later. We observed increased prolongations of the time from symptom onset until hospital admission and the door-to-balloon time. The systemic delay in the door-to-balloon time could be explained by the need for more detailed protocols to identify suspected cases of COVID-19 and the need for additional staff preparation involving the donning of personal protective equipment.

The increased rate of PCI in NSTEMI patients in 2020 can be linked to the increased incidence of acute heart failure, which affected the need for immediate PCI. The treatment strategy and PCI rates remained unchanged in STEMI patients. In the United States, Garcia et al. [11] reported a reduction in activations for primary PCI for STEMI of 38%. Piccolo et al. [3] reported a 32% reduction in the number of PCIs across the entire spectrum of ACS patients in Italy; in Spain, Rodríguez-Leor et al. [12] noted a 40% reduction in activations for primary PCI for STEMI.

The exact causes of the decreased number of admitted ACS patients are unknown. However, fear of hospitals as a place where people could potentially be infected and ignoring the signs and symptoms of myocardial ischaemia and mistaking them for the signs and symptoms of respiratory infections surely resulted in patient-related delays and a reduced number of hospitalisations. A more sedentary lifestyle may have influenced reduced hospital admissions related to UA.

Patients presented with higher complication and mortality rates in our study. Current data on the mortality rates of ACS patients during the COVID-19 outbreak are scarce.

Metzler et al. [9] hypothesised that cardiovascular mortality due to untreated ACS is negatively affected by patients not coming to the hospital. De Rosa et al. [1] reported a substantially increased STEMI fatality rate and major complications during the pandemic, while Secco et al. [2] reported no difference in in-hospital mortality before and during the COVID-19 pandemic.

In this study, we found significantly decreased hospitalisation duration in 2020, which can be explained by the early discharge of stable patients as one of the hospital measures implemented to avoid hospital-acquired infections in those patients.

Ignored and unrecognised myocardial-ischaemia-related symptoms may cause missed and delayed hospital admissions and increase short- and long-term complications and mortality in ACS patients. Restrictive measures were very effective in limiting the spread of the virus but resulted in a dramatic reduction in hospital admissions of ACS patients. Although we had no SARS-CoV-2-infected patients with ACS, we still observed markedly increased mortality in STEMI patients. Time is a very important factor in preventing myocardial-infarction-related complications. Even given the restrictive measures implemented to control the COVID-19 outbreak, special consideration needs to be given to patients with symptoms of myocardial ischaemia and protocols need to be implemented, in order to reduce morbidity and mortality related to missed or delayed treatment of acute cardiovascular diseases such as ACS. Timely diagnosis and in-hospital treatment of these ACS patients are an important public and health care responsibility. Patients should not ignore myocardial-ischaemia-related symptoms, and educating the public with regard to the recognition and diversity of symptoms and the benefits of presenting promptly to the hospital or emergency ambulance service is of great importance.

## Conclusion

A decrease in the number of patients hospitalised for ACS and prolonged ischaemic duration during the COVID-19 outbreak in Serbia contributed to the increased complications and mortality in these patients. These findings highlight the potentially harmful consequences of restrictive measures on other acute conditions, such as ACS. Thorough assessment before the proposal and implementation of restrictive measures is needed in the future.
